# Prevalence and geographical distribution of persons with albinism (PWA) in Togo in 2024

**DOI:** 10.1371/journal.pone.0360507

**Published:** 2026-10-14

**Authors:** Malanna Bigma, Kparakate Kota-Mamah, Rodion Yao Konu, Marc Harris Dassi Tchoupa Revegue, Christophe Przybylski, Souradj Our-Yendo, Palokinam Pitche, Didier Ekouevi, Bayaki Saka

**Affiliations:** 1 African Center for Research in Epidemiology and Public Health, Lomé, Togo; 2 Department of Public Health, University of Lomé, Lomé, Togo; 3 Training and Research Center in Public Health, Lomé, Togo; 4 Scientific Direction, Pierre Fabre Foundation, Lavaur, France; 5 National Association of Persons with Albinism in Togo, Lomé, Togo; 6 Togolese Society of Dermatology and Sexually Transmitted Infections (SOTODERM), Lomé, Togo; Bogomolets National Medical University: Nacional’nij medicnij universitet imeni O O Bohomol’ca, UKRAINE

## Abstract

**Background:**

Persons with albinism (PWAs) in Africa represent a vulnerable population facing social discrimination, severe health issues such as skin cancers, and precarious socioeconomic conditions. The objective of this study was to estimate the prevalence of albinism and map the distribution of persons with albinism in Togo in 2024.

**Method:**

This cross-sectional descriptive study was conducted from November 2023 to October 2024 across all 39 health districts in Togo, and it included a telephone survey in which community health workers (CHWs) identified PWAs in their districts. CHWs were subsequently trained to perform onsite data collection using the Kobocollect application. Finally, the data were reviewed by national association representatives of PWAs to validate the data and address any gaps. The data were analysed using R software, while geographic mapping was performed with QGIS.

**Results:**

A total of 1,521 PWAs were identified in Togo, which is a prevalence of 18.7 PWAs per 100,000 inhabitants (equivalent to 1/5,323; 95% CI [1/5,605; 1/5,065]). Sociodemographic and regional characteristics were available for 1,433 PWAs who consented to participate in this study and were included in the PWA database in Togo. Districts such as Kpélé, Danyi, and Amou presented the highest density of PWAs. The PWA population is predominantly young, with 62.7% under 25 years of age, and it has a slight female predominance (52.2%). Approximately 70.7% of PWAs reported daily exposure to the sun without adequate protection. Moreover, 61.6% had no regular income, and only 1.4% earned over 100 000 FCFA per month. Over 30% of PWAs experienced health issues related to albinism, yet only 37% had a medical follow-up record.

**Conclusion:**

This first national study in Togo allowed us to estimate the prevalence of albinism in the country. The geographic distribution shows that PWAs are present throughout Togo, with concentrations in large cities. This type of research must continue to enable early identification and monitoring of PWAs to reduce the occurrence of serious health problems such as skin cancers.

## Introduction

Albinism is a rare genetic condition characterized by the partial or complete absence of melanin, the pigment responsible for skin, hair, and eye colour. It is caused by mutations in genes responsible for melanin synthesis, most commonly in the TYR gene, which encodes tyrosinase, an enzyme critical to melanin production [1]. Albinism manifests in various forms, including oculocutaneous albinism (OCA) and syndromic types, such as Hermansky–Pudlak syndrome and Chediak–Higashi syndrome, which present additional systemic complications [2].

In Africa, albinism is more common than in many other parts of the world, with prevalence estimates ranging from 1/5,000–1/1,000 depending on the population studied [3]. High prevalence rates have been reported in Zimbabwe and Tanzania, attributed to genetic bottlenecks and cultural practices of endogamy in certain communities [4]. However, many African countries, including Togo, lack comprehensive data on the prevalence and distribution of persons with albinism (PWAs). This scarcity of data hinders the development of targeted interventions and policies to address health and social challenges. PWAs face significant health risks due to their reduced melanin levels, which provide limited protection against ultraviolet (UV) radiation. This makes them particularly susceptible to skin damage, including sunburn, precancerous lesions, and skin cancers such as basal cell carcinoma and squamous cell carcinoma [5]. Studies in sub-Saharan Africa indicate that skin cancers are a leading cause of morbidity and mortality among PWAs, with a median age of diagnosis of approximately 30–40 years [3]. Additionally, many PWAs suffer from visual impairments due to structural abnormalities in the retina and optic nerve development caused by the absence of melanin [4]. In addition to health issues, PWAs experience pervasive social stigma and discrimination, which are often rooted in cultural beliefs and myths. In some communities, albinism is associated with supernatural explanations, leading to ostracism, violence, and, in extreme cases, ritual killings [3]. Indeed, the social inclusion of persons with albinism remains hindered by persistent stigma and discrimination. In addition, limited economic and employment opportunities mean that, because of unemployment and poverty, many are compelled to undertake occupations that expose them to substantial health risks, including agricultural work, street vending, and masonry under prolonged exposure to sunlight [6].

In Togo, the literature on albinism has focused primarily on small urban populations, such as studies conducted in the capital, Lomé [5]. These studies reported high levels of stigma, poor access to health care, and alarming rates of skin cancers among PWAs. However, no nationwide epidemiological study has been conducted to estimate the prevalence of albinism or map its geographical distribution in Togo. This study aimed to estimate the prevalence and geographic distribution of PWAs in Togo.

## Methods

### Study framework and period

Togo is a West African country covering an area of 56,600 km^2^, with an estimated population of 8,095,498 according to the latest general population census conducted in 2022 [7]. This population is predominantly young and resides mainly in rural areas. The poverty rate is significantly higher in rural areas (58.8%) than in urban areas (26.5%) [8]. The organization of the Togolese health care system is based on a three-tiered pyramid structure: central (ministry and central directions), intermediate (regional health directions), and peripheral (prefectural health directions). Togo has 39 prefectural directions for managing local health services. In 2022, the country had 2,152 health care facilities to meet the curative, preventive, and promotional health needs of its population [9]. This study was conducted from November 2023 to October 2024.

### Study population

The study targeted all PWAs residing in Togo, defined as those with visible characteristics such as light-coloured skin, hair, and eyes consistent with oculocutaneous albinism [10]. The participants were identified by community health workers (CHWs) and representatives from the national association of persons with albinism in Togo.

### Data collection

#### Data collection occurred in three systematic phases.

**Phase 1: Reporting by community health workers via phone calls:** This phase was only declarative and aimed to collect information on the presence and number of PWAs within the intervention areas of each CHW. Two call centres were established: one in Lomé to cover the regions of Grand Lomé, Maritime, and Plateaux and the other in Kara for the Centrale, Kara, and Savanes regions. Each centre recruited and trained 50 agents, each responsible for contacting 75 CHWs using lists of phone numbers. In total, 7,500 active CHWs in Togo were contacted. In large cities, community relays were also mobilized to identify PAA in their respective areas.

**Phase 2: Case identification and data collection by community health workers (CHWs):** The second step of the process was preceded by a cascade training of CHWs. The main purpose of this phase was to ensure a good identification of PWA cases This began with the training of district delegates, which was conducted in regional capitals. The training was led by the project coordination team, consisting of two physicians (the project manager and a clinical study monitor), a biostatistician, and the president of the association for persons with albinism in Togo. District delegates, appointed by the district directors of health, received comprehensive training on the survey, its objectives, the tasks of the investigators, and the data collection tool. When they returned to their districts, the district delegates trained the CHWs, equipping them with the necessary skills to understand the study objectives, identifying persons with albinism in their areas, using the data collection tool effectively, and administering the digitalized questionnaires. The questionnaire used for this purpose was designed specifically for the present study.

**Phase 3: Completeness check with representatives of the national association of persons with albinism:** The district representatives of the national association of PWAs in each region and district of Togo were mobilized. This phase was carried out in two steps, preceded by a cross-analysis of data between those collected by community health workers and the database of the national association of persons with albinism in Togo. This analysis helped identify PWAs who had already been surveyed and provided the prefectural presidents with an opportunity to complete missing data for PWAs not yet registered, using the PWA registry that each prefectural president possesses. The first step involved training the prefectural chairperson on data collection tools, particularly the use of Kobocollect and information retrieval from the registries.

#### Addressing duplicates.

As part of this study, some efforts were made to avoid or eliminate duplicates. To achieve this, the pseudonymization technique was employed. Each CHW conducting the survey was provided with a list of pseudonyms prepared by the CARESP team. The CHW assigned a pseudonym to each surveyed PWAs. The pseudonyms were digitized and categorized by region and district, allowing the CHW to select the appropriate pseudonym on the basis of the assigned code after verification against the provided list. Finally, a manual filtering process was carried out independently by two researchers from the study team on the surnames and first names and then on variables such as age, district of birth, and district of residence to detect and delete duplicates.

### Data analysis

The quantitative data were analysed using R software (R version 4.4.1 (2024-06-14 urct)). Descriptive statistics were generated to calculate frequencies, proportions, and prevalence rates. For geographic analysis, QGIS 3.8.1 software was used to map the spatial distribution of PWAs, identifying clusters and regions with relatively high densities. Prevalence estimates were calculated using population data from the 2022 national census [9].

### Ethical considerations

The study was approved by the Togolese Committee for Bioethics and Research in Health (Approval No. 049/2023/CBRS). Written informed consent was obtained from all participants or their legal guardians. The anonymity of the participants was maintained throughout the study.

## Results

### Prevalence of albinism in Togo

A total of 1,521 PWAs were identified in Togo. Among the identified PWAs, 88 (5.78%) refused to be registered in the database. The number of PWAs identified in this survey represents a prevalence of 18.79 PWAs per 100,000 inhabitants (equivalent to 1/5,323; 95% CI [1/5,605; 1/5,065]). We noted a lack of data for the 88 PWAs who refused to be registered. Therefore, we present the results on the distribution and sociodemographic characteristics of the 1,433 PWAs for whom we obtained complete data.

On the basis of the population in each region, a high proportion of PWAs is observed in the Plateaux region, with 40 PWAs per 100,000 inhabitants, whereas the lowest proportion is in the Savanes region, North region, with 9 PWAs per 100,000 inhabitants (Table 1).

**Table 1:** Proportion of PWA in each region of Togo in 2024.

	Population	Number of PWA	‰
**Savanes**	1 143 520²	180	0.09
**Kara**	985 512	227	0.20
**Centrale**	795 529	200	0.14
**Plateaux**	1 635 946	390	0.40
**Maritime**	1 346 615	164	0.21
**Grand Lomé**	2 188 376	272	0.16

The proportion of PWAs relative to the population varies according to health district. The districts of Kpélé and Danyi (40 PWAs per 100,000 inhabitants) and Amou (37 PWAs per 100,000 inhabitants) in the Plateaux region were the districts with the highest PWAs density in Togo. These are followed by the districts of Mô (34 PWAs per 100,000 inhabitants) in the Centrale region and Kozah (33 PWAs per 100,000 inhabitants) in the Kara region. The lowest proportions are found in the districts of Avé in the maritime region and Akébou in the Plateaux region, each with 1 PWA per 100,000 inhabitants (Table 2).

**Table 2:** Proportion of PWA in each district in Togo in 2024.

	Population	Number of PWA	‰
**Savanes**			
**Cinkassé**	128959	22	0.17
**Kpendjal**	88365	10	0.11
**Kpendjal ouest**	123330	16	0.13
**Oti**	124848	20	0.16
**Oti sud**	150376	22	0.15
**Tandjouaré**	138867	20	0.14
**Tône**	388775	70	0.18
**Kara**			
**Assoli**	66394	18	0.27
**Bassar**	152065	25	0.16
**Binah**	84199	21	0.25
**Dankpen**	185662	39	0.21
**Doufelgou**	84767	15	0.18
**Kéran**	128687	16	0.12
**Kozah**	283738	93	0.33
**Central**			
**Blitta**	163272	20	0.12
**Mô**	52448	18	0.34
**Soutouboua**	138864	28	0.20
**Tchamba**	200585	69	0.34
**Tchaoudjo**	240360	65	0.27
**Plateaux**			
**Agou**	85873	7	0.08
**Akebou**	73830	1	0.01
**Amou**	114172	42	0.37
**Anié**	180158	58	0.32
**Danyi**	40240	16	0.40
**Est mono**	164460	37	0.22
**Haho**	305096	56	0.18
**Kloto**	145986	38	0.26
**Kplélé**	80939	32	0.40
**Moyen mono**	90505	20	0.22
**Ogou**	253467	54	0.21
**Wawa**	101300	29	0.29
**Maritime**			
**Avé**	111214	1	0.01
**Bas mono**	94860	14	0.15
**Lacs**	241247	30	0.12
**Vo**	224411	15	0.07
**Yoto**	174851	27	0.15
**Zio**	500032	77	0.15
**Grand Lomé**			
**Agoè nyivé**	882695	125	0.14
**Golfe**	1305681	147	0.11

### Sociodemographic characteristics of PWAs

The median age of the identified and registered PWAs was 18 years (IQR [9–34]). The PWA population in Togo is predominantly young, with 62.7% under the age of 25 (Table 3). Infants and adolescents aged 1–15 years make up 43.6% of PWAs, whereas those over 45 years represent 11.2%. Female PWAs accounted for 52.2% of the population studied. Among all PWAs residing in Togo, 98.4% were Togolese, while 1.0% originated from neighbouring countries (Benin, Burkina Faso, Ghana, and Côte d’Ivoire).

**Table 3:** Socio-demographic characteristics of PWA in Togo in 2024.

	Number (n)	Proportion (%)
**Age (years)**		
Mean (Ecart-Type)	23 (18)	
Median [IIQ]	18 [9-34]	
**Age (years)**		
<15	624	43.60
[15–24]	276	19.30
[25–34]	218	15.20
[35–45]	153	10.70
≥ 45	161	11.20
**Sex**		
Female	755	52.70
Male	678	47.30
**Nationality**		
Togolese	1 408	98.30
Other	24	1.70

IIQ = interval interquatil.

With respect to lifestyle, 70.6% of the surveyed PWAs reported engaging in activities that involved daily sun exposure for more than two hours without adequate protection. Among the PWAs, over half (54.8%) were members of an association for PWAs, and 30.7% reported health issues, particularly skin lesions related to albinism (Table 4). From a socioeconomic perspective, 61.6% of PWAs had no income activity. This group primarily consisted of children, students, or unemployed adolescents and young adults. Among the PWAs who were employed, 73,09% earned a monthly income of less than 75 euros, for reference, in Togo, as of January 1, 2023, the guaranteed minimum wage and agricultural minimum wage are 80 euros per month [11]. Only 3.8% lived modestly, with an income exceeding 150 euros.

**Table 4:** Lifestyle and socio-economic conditions of PWA in Togo 2024 (N = 1,433).

	Number (n)	Proportion (%)
**Sun exposure >2 h**		
No	421	29.40
Yes	1 012	70.60
**Membership of the national association of PWA**		
No	648	45.20
Yes	785	54.80
**Health problems related to albinism**		
No	1 003	70.00
Yes	430	30.00
**Having a follow-up booklet**		
No	919	64.10
Yes	514	35.90
**Monthly income (in Euro)**		
Not applicable	883	61.60
0 < 75	402	28.00
[75–115]	89	6.20
[115 −150]	38	2.70
> 150	21	1.50

### Geographic distribution of PWAs in Togo

PWAs are present throughout Togo; however, the plurality were identified in the Plateaux region (27.91%) (Fig 1). The projection of geolocation points allowed us to observe a homogeneous distribution of PWAs across the entire country, with concentrations of PWAs in large cities (Fig 1).

**Fig 1:**
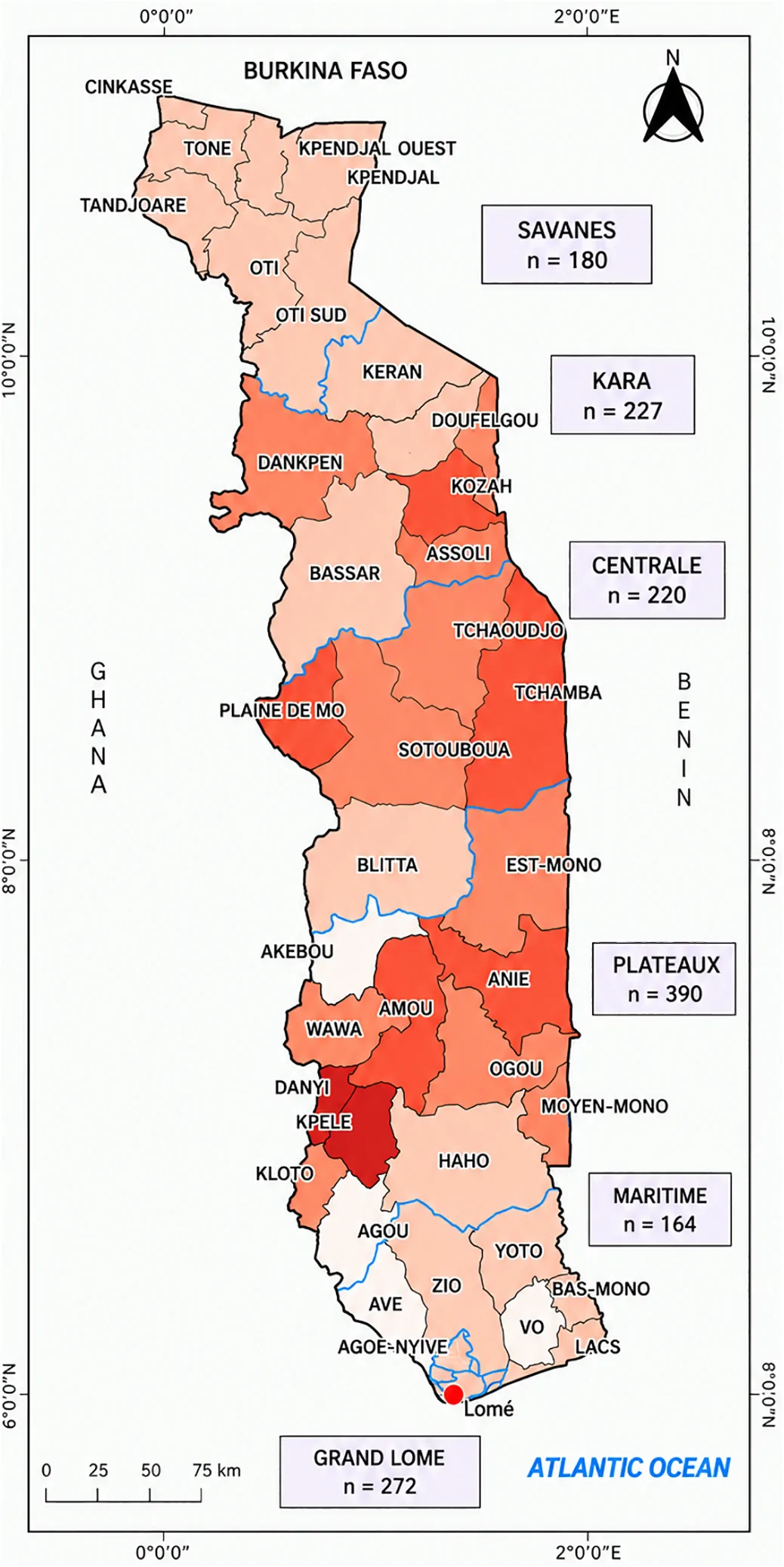
Distribution of PWA in Togo. **Legend:** The map shows the geographical distribution of persons with albinism in Togo. The colour gradient represents the density of persons with albinism per 100,000: very light gray: 0.01–0.09; very light beige: 0.10–0.19; light orange: 0.20–0.29; dark orange: 0.30–0.39; and orange-red: > 0.40 per 100,000 inhabitants. Light blue outlines indicate the boundaries of the health regions Distribution of PWA in Togo, 2024, made using QGIS © 2024 by BIGMA Malanna is licensed under CC BY 4.0. To view a copy of this license, visit https://doi.org/10.5281/zenodo.22831197.

In each health region, a concentration of PWAs is observed in the regional capitals. The proportion of PWAs residing in regional capitals is the same as that of the general population; the majority of the population resides in regional capitals (Fig 1).

The population of PWAs residing in the districts varied. The districts of Danyi, Kpélé, and Amou in the Plateaux region had high densities, with at least 42 PWAs per 100,000 in each of the districts (Fig 1).

## Discussion

### Key findings

The important findings of this study show that: (i) The national prevalence of albinism was estimated at 1 in 5,323 individuals (95% CI: [1/5,605–1/5,065]); (ii) A significant proportion of the PWAs consists of children and adolescents, with 44% under the age of 15; (iii) Three out of four PWAs reported a monthly income below 75 euros; (iv) The Plateaux Region showed the highest prevalence three districts of of Danyi, Kpélé, and Amou with particularly high densities of PWAs.

### Validity and limitations

The strengths of this study include that, to our knowledge it is the first nationwide assessment of albinism prevalence in Togo. It combined multiple data sources, including the active identification of persons with albinism by community health workers. The study also involved the national association of persons with albinism in Togo. Its regional and district representatives helped verify and supplement the data using their registers.

The limitation of this study is the absence of a qualitative component to explore the experiences of PWAs in Togo. Additionally, diagnosing albinism was sometimes challenging, as some individuals identified as having albinism may have been “red-haired persons” rather than confirmed PWAs. Although the community health workers (CHWs) were thoroughly trained in identifying persons with albinism, dermatologists did not confirm the identification, which may introduce a potential bias in case identification. However, this bias was mitigated by the training provided to the CHWs, as well as the support of the study’s physicians, representatives, and members of the national association of persons with albinism in Togo. The reported prevalence may underestimate the true prevalence due to possible under-ascertainment related to stigma, reluctance to disclose status, and limited identification of marginalized or geographically isolated individuals. Additionally, sustained awareness campaigns are needed to strengthen the ability of PWAs to adopt good health practices and seek early medical consultations. These measures could help reduce the incidence of severe health conditions related to albinism, such as skin cancer.

### Prevalence of albinism in Togo

The prevalence of 18.79 PWAs per 100,000 inhabitants in Togo falls within the range reported in sub-Saharan Africa but shows regional variations. Studies in Nigeria indicate a prevalence ranging between 15 and 30 PWAs per 100,000 inhabitants [3]. However, certain regions report higher rates due to genetic and environmental factors. For example, albinism in sub-Saharan Africa is influenced by the distribution of genetic mutations and sociocultural practices that may cluster cases in specific communities [12]. Our findings with regard to the prevalence of PWAs in Togo, in West Africa, are similar to findings reported in East Africa, particularly in Tanzania, where the prevalence of albinism is estimated at approximately 20 PWAs per 100,000 inhabitants [3].

### Geographic distribution of PWAs

The geographic distribution of PWAs in Togo reveals a nationwide presence, with a significant concentration in the Plateaux region (27.91%). This clustering aligns with trends observed in other African countries, where PWAs gravitate toward regions with better access to resources, health care, and education. For example, in Malawi and Tanzania, urban and peri-urban areas show higher concentrations of PWAs because these areas contain more economic opportunities and safety from stigma compared with rural areas [13,14]

### Sociodemographic characteristics and living conditions

The data revealed that 62.78% of PWAs in Togo are under the age of 25, indicating a predominantly young population. This finding is consistent with a study in Brazil in which 57.7% of PWAs were between 18 and 40 years old [13]. The young demographic profile may reflect both the general population structure in these regions and the limited life expectancy of PWAs, which is often associated with a lack of health care access, vulnerability to skin cancer, and socioeconomic challenges. Public health strategies targeting persons with albinism (PWAs) should explicitly address the specific challenges associated with childhood and adolescence, and ideally be implemented early in the life course.

In Togo, 61.6% of PWAs had no income activity, whereas only 3.8% earned more than 100,000 FCFA per month. This highlights significant socioeconomic vulnerabilities among PWAs, particularly in terms of employment and income. These findings are consistent with studies in other African countries that document systemic inequalities faced by PWAs, such as limited access to education, health care, and employment opportunities. A study conducted in Harare, Zimbabwe revealed that biological constraints that characterize persons with albinism negatively impact their prospects of becoming employed and remaining employed. Furthermore, discrimination against persons with albinism has led to challenges in participating in the workforce [13–15].

## Conclusion

This first national study in Togo allowed us to estimate the prevalence of albinism in the country. The geographic distribution shows that persons with albinism are present throughout Togo, with high concentrations in large cities. This first nationwide study on albinism in Togo provides essential data on the prevalence and distribution of persons with albinism. The findings will serve as a foundation for conducting targeted studies to better understand the specific needs of this population. They will also contribute to improving the care and support available to persons with albinism, guiding prevention and awareness efforts. Ultimately, these actions aim to reduce complications associated with albinism, such as skin cancer, and improve the overall well-being of affected individuals.

## Supporting information

S1 File Questionnaire mapping of persons with albinism. (DOCX)
