# Supplementary material for: Prevalence and geographical distribution of persons with albinism (PWA) in Togo in 2024
Source: PLoS One. 2026 Oct 14;21(10):e0360507. doi: 10.1371/journal.pone.0360507 (PMC13667812; doi:10.1371/journal.pone.0360507)
Supplement: Supplementary file 1 [file pone.0360507.s001.docx]

**Mapping of Persons with Albinism (PWA) in Togo in 2024**

***Important information to read before filling out the questionnaire***

*All collected information will be treated strictly anonymously and only used for this study.*

*Take time to read the questions. Tick the answers that best match the responses.*

***Note: this questionnaire is implemented on KoboCollect, and the electronic version will be used for data collection via interviewers' smartphones.***

Date of data collection: I___I___I/ I___I___I/ I___I___I___I___I

|  | **Variables / Questions** | **Options** |
| --- | --- | --- |
| **Location** | | |
| 1. | Health regions | ❑ Grand Lomé ; ❑ Maritime ; ❑ Kara ;  ❑ Centrale ;❑ Plateaux ; ❑ Savanes |
| 2. | Health districts | Set by region (Health districts show up according to the health region chosen in 1) |
| 3. | Interviewer ID | A drop-down list of interviewers’ ID |
| 4. | Geolocation | ………………………………… (The geolocation point to be automatically captured by the smartphone) |
| **Socio-demographic characteristics of the PWA** | | |
| 5. | Unique participant ID | A drop-down list of participants ID set according to the location and the interviewer |
| 6. | Age (years) | I___I___I___I |
| 7. | Birth district | A drop-down list of all 39 districts (in alphabetical order) |
| 8. | Sex | ❑Male ❑ Female |
| 9. | Nationality | ❑Togolese ❑ Other, specify ………………… |
| **Lifestyle** | | |
| 10. | Are you often (more than two hours) exposed to the sun during the day without adequate protection?  *A hat does not count as adequate protection in this case* | ❑ Yes ❑ No |
| 11. | Are you registered in an association for persons with albinism? | ❑ Yes ❑ No |
| 12. | Are you being monitored for a health problem related to albinism? | ❑ Yes ❑ No |
| 13. | Do you have a follow-up booklet for albinism? | ❑ Yes ❑ No |
| 14. | Monthly income (in EURO) | ❑ Not applicable  ❑ <75  ❑ ]75 – 115]  ❑ ]115 – 150]  ❑ > 150 |
| 15. | The respondent (PWA) has at least one lesion on the body | ❑ Yes ❑ No |
| **Sharing of personal information (optional)** | | |
| 16. | Would you like to share your personal data (Last name, first name, and phone number) to build the PWA database in Togo? | ❑ Yes ❑ No  If no, end of questionnaire |
| 17. | Last name | ………………………… |
| 18. | First name(s) | …………………………………………………… |
| 19. | Phone number  *Format = 00xxx xx xx xx xx* | …………………………. |
