# Supplementary material for: Prevalence and geographical distribution of persons with albinism (PWA) in Togo in 2024
Source: PLoS One. 2026 Oct 14;21(10):e0360507. doi: 10.1371/journal.pone.0360507 (PMC13667812; doi:10.1371/journal.pone.0360507)
Supplement: Supplementary file 2 [file pone.0360507.s003.docx]

# Response to Reviewers

| N° | Questions | Response |
| --- | --- | --- |
|  | **Reviewer #1** | |
| 1 | Reference 2 should be revised. Instead of providing a web link to the textbook, the authors should cite the book according to the journal style, including the relevant edition and page numbers | Done:  **New**: Grob JJ, Saurat JH, Grasshans E, Lachapelle JM, Lipsher D, Thomas L, Lacour JP, Naeyaert JM, Salemon D. Dermatologie et Infections Sexuellement Transmissibles. |
| 2 | Reference 7 is currently inappropriate because it refers to a Google search result. The authors should replace it with the original ANAT (Association Nationale des personnes Atteintes d'Albinisme au Togo) document and provide a proper citation. The content derived from this source should also be more clearly described in the manuscript. | Done:  **New**: Plan-strategique-pour-l'inclusion-socioéconomique-des-personnes-atteintes-d'albinisme-au-togo-psiaT.pdf. visit: <https://anat.tg/wp-content/uploads/sites/4/2020/08>. |
| 3 | In the Results section, the statement "Among the PWAs who were employed, 73.09% earned a monthly income of less than 75 euros (for reference, in Togo, as of January 1, 2023, the guaranteed minimum wage and agricultural minimum wage are 80 euros per month)" should be supported by an appropriate governmental or institutional reference. | Done:  Reference added : RTG. SMIG et SMAG : le gouvernement acte les nouveaux montants. Site officiel du Togo, République Togolaise, https://www.republiquetogolaise.tg/gestion-publique/0401-7605-smig-et-smag-le-gouvernement-acte-les-nouveaux-montants (accessed 6 August 2026) |
| 4 | In the Discussion section, the sentence "This finding is consistent with a study in Brazil in which 57.7% of PWAs were between 18 and 40 years old" requires an explicit reference, which is currently missing | Done:  This actually is the reference 14:  Maia M, Volpini BMF, Santos GA dos, et al. Quality of life in patients with oculocutaneous albinism. An Bras Dermatol 2015; 90: 513–517. |
| 5 | Table 4 reports that 30% of participants experienced health problems related to albinism. The manuscript would benefit from a more detailed description of these conditions in the Results section. If available, the authors should specify the most common health problems observed | Detailed data on the various health manifestations associated with albinism were not collected because this was beyond the specific objectives of the present study. |
| 6 | The discussion of the geographical distribution could be expanded. Additional discussion of possible genetic, demographic, healthcare-access, migration, or sociocultural factors contributing to this distribution would strengthen the interpretation of the findings | Thanks for the suggestion, I am taking it into consideration |
|  | **Reviewer #2** | |
| 7 | This is a valuable epidemiological study addressing an important public health issue. The authors should be commended for conducting the first nationwide survey of persons with albinism in Togo. The dataset is substantial, the methodology is appropriate for the study objectives, and the manuscript is generally well written. The findings will likely be useful for healthcare planning and future research in the region | Thanks |
| 8 | However, the manuscript is largely descriptive. It provides prevalence estimates and geographic mapping but offers limited analytical depth or broader scientific insight. The discussion remains relatively brief and could better place the findings in an international context. Expanding the interpretation of the results and their implications would further strengthen the manuscript. | Thanks for the suggestion, I am taking it into consideration |
