# Supplementary material for: Prevalence and geographical distribution of persons with albinism (PWA) in Togo in 2024
Source: PLoS One. 2026 Oct 14;21(10):e0360507. doi: 10.1371/journal.pone.0360507 (PMC13667812; doi:10.1371/journal.pone.0360507)
Supplement: Supplementary file 3 [file pone.0360507.s004.docx]

Dear Reviewers,

We have carefully reviewed your comments, suggestions, and questions regarding our manuscript **“PONE-D-26-17595R1, Prevalence and geographical distribution of persons with albinism (PWA) in Togo in 2024”**. We sincerely thank you for your valuable and constructive feedback, which has helped us improve the quality and clarity of our manuscript.

In response to your comments and questions, please find in the attached response table our detailed answers, along with the corresponding modifications made to the manuscript.

Thank you again for your time and thoughtful review.

Sincerely,

**Dr BIGMA Malanna**

# Response to Reviewers

| N° | Questions | Response |
| --- | --- | --- |
| 1 | A minor comment pertains to the authors acknowledging in the limitations that the reported prevalence may underestimate the true number of persons with albinism in the study area. Given the documented stigma and social discrimination surrounding albinism in Togo and the broader West African context, some individuals or families may be reluctant to self-identify or disclose their status, even when community health workers are involved in case identification. In addition, socially marginalized or geographically isolated individuals may be less likely to be identified through community-based enumeration. The authors should acknowledge this potential under-ascertainment and exercise caution in interpreting the reported figures as an exact prevalence estimate. Rather, the estimates should be presented as reflecting the number of persons with albinism identified through the study's ascertainment approach | In the limits section  The reported prevalence may underestimate the true prevalence due to possible under-ascertainment related to stigma, reluctance to disclose status, and limited identification of marginalized or geographically isolated individuals |
